# Release, Cytocompatibility, and In Vivo Biocompatibility of WJ-MSC-Supernatant-Loaded PLGA/NMP In Situ-Forming Implants

**DOI:** 10.3390/jfb17100507

**Published:** 2026-10-07

**Authors:** María Concepción Álvarez-García, Gabriela Piñón-Zárate, César Beato-López, Laisa Ximena Contreras-Aguilar, Andrés Eliú Castell-Rodríguez, Sara Judith Álvarez-Pérez, Erick Francisco Sánchez Ortega, Alin García-Miranda, Jorge Esteban Miranda-Calderón, Katia Jarquín-Yáñez

**Affiliations:** 1 Departamento de Periodoncia, Facultad de Odontología, Universidad Nacional Autónoma de México, Ciudad de México C.P. 04510, Mexico; 2 Departamento de Biología Celular y Tisular, Facultad de Medicina, Universidad Nacional Autónoma de México, Ciudad de México C.P. 04510, Mexico; 3 Laboratorio Internacional EPIGEN-BUAP, Centro de Química Instituto de Ciencias (ICUAP), Benemérita Universidad Autónoma de Puebla, Puebla C.P. 72570, Mexico; 4 Departamento de Sistemas Biológicos, Universidad Autónoma Metropolitana, Calzada del Hueso 1100, Colonia Villa Quietud, Alcaldía Coyoacán, Ciudad de México C.P. 04960, Mexico

**Keywords:** mesenchymal-stem cells, PLGA, in situ-forming implants, cytotoxicity

## Abstract

(1) Background: Modern pharmacology and regenerative medicine seek effective, safe drug delivery systems that allow for the controlled and sustained release of active ingredients; in situ forming implants (ISFIs) represent one such option. Systems based on poly(lactic-co-glycolic acid) (PLGA) and the solvent *N*-methyl-2-pyrrolidone (NMP) offer a safe, biodegradable, and non-invasive alternative. (2) Methods: ISFIs were prepared using NMP and PLGA at 50:50 and 85:15 ratios, combined with the supernatant obtained from umbilical cord Wharton’s jelly-derived mesenchymal stem cells (WJ-MSCs). Cellular utility, cytotoxicity, cell proliferation, and in vivo biocompatibility were evaluated using Annexin, 7AAD, Calcein, and PrestoBlue assays, as well as a murine model. Statiscal significance was determined using one way ANOVA followed by Tukey’s post hoc test for multiple comparisons, with the significance level at *p* < 0.05. (3) Results: Differences in cumulative release profile were observed between the PLGA/NMP 50:50 and 85:15 formulations. PLGA 50:50 exhibited higher early and cumulative release of WJ-MSC-supernatant-derived material, whereas PLGA 85:15 showed a slower and more sustained release profile. (4) Conclusions: Cell-free therapy delivered via PLGA 50:50/NMP ISFIs, incorporating associated immunomodulatory factors as supernatant’s WJ-MSCs, is suitable for an effective controlled-release system.

## 1. Introduction

Parenteral controlled-release systems have emerged as a relevant pharmacological strategy. They maintain constant plasma concentrations of the active ingredient over extended periods and minimize plasma peaks and troughs that compromise treatment safety and efficacy [1,2,3]. Among available systems, in situ-forming implants (ISFI) have emerged as a relevant approach. They can release the active ingredient over days or even months without requiring surgical implantation [4,5]. In an injectable controlled-release system, poly(lactic-co-glycolic acid) (PLGA) is the predominant synthetic biodegradable polymer, owing to its well-established toxicological profile and FDA approval for many therapeutic applications [3,6,7]. PLGA is a copolymer composed of lactic acid and glycolic acid units. The ratio of these monomers, the molecular weight, and the nature of the terminal group (acid or ester) directly influence hydrolytic degradation and the release kinetics of the encapsulated active ingredient [6,7]. These physicochemical properties, combined with its capacity to modulate drug release from days to over six months, have established PLGA as the material of choice for long-acting parenteral formulations, as seen in commercial products such as leuprolide acetate (Lupron Depot^®^, Eligard^®^), risperidone (Risperdal Consta^®^), or goserelin (Zoladex^®^) [1,5,8].

ISFI consists of a liquid polymer solution in a water-miscible organic solvent containing the dissolved or dispersed drug. Following subcutaneous or intramuscular administration, the solvent diffuses into surrounding biological fluids while water penetrates the polymeric solution, triggering phase inversion that transforms the solution into a solid or semisolid depot in situ [4,9]. *N*-Methyl-2-pyrrolidone (NMP), a water-miscible organic solvent, is the most commonly used in PLGA-based ISFI formulations. This water miscibility is essential for appropriate implant formation kinetics [5,10]. The Atrigel^®^ system, used in commercial formulations, is a PLGA solution in NMP, highlighting the clinical relevance of this solvent system [11]. Although NMP is an FDA-approved pharmaceutical excipient, its parenteral administration raises significant toxicological concerns. The rapid, extensive diffusion of NMP into surrounding tissues during the initial hours following injection constitutes the primary mechanism underlying early-phase cytotoxicity. This effect is linked to local tissue irritation, injection-site swelling, and a high initial drug release (burst effect), which may compromise safety and efficacy [5,9,10]. Cytotoxicity studies have shown that direct exposure of viable cells to NMP reduces cell viability in a concentration- and time-dependent manner. Although previous studies have described the toxicological behavior of NMP in ISFI, biocompatibility must still be specifically evaluated for each formulation because the biological response depends on the exact polymer composition, solvent load, and release kinetics. In PLGA/NMP-based ISFI, the local exposure to solvent during phase inversion, together with the acidification associated with PLGA degradation, can modify cell viability and inflammatory response [10].

Alongside advances in controlled-release systems, regenerative medicine has grown rapidly driven by the discovery of the therapeutic potential of mesenchymal stem cells (MSCs), particularly those from Wharton’s jelly of the umbilical cord (WJ-MSCs) [12,13]. WJ-MSCs are non-hematopoietic multipotent cells found in the extracellular matrix around umbilical cord vessels. Notably, this source, considered medical waste, avoids the ethical concerns associated with other cell sources [12,14]. Furthermore, compared with MSCs derived from bone marrow or adipose tissue, WJ-MSCs exhibit higher in vitro proliferation rates, reduced cellular senescence, superior genomic stability, a more favorable immunological profile, and enhanced expression of pluripotency markers such as Nanog, Oct3/4, and Sox2 [12,15]. However, evidence from the past decade suggests the main therapeutic effect of WJ-MSCs is their strong paracrine activity. This occurs through the secretion of bioactive molecules collectively known as the secretome [12,16,17]. The WJ-MSC secretome includes soluble factors, such as cytokines, chemokines, growth factors, angiogenic mediators, prostaglandins, and extracellular vesicles [18]. These extracellular vesicles are exosomes and microvesicles that carry functional proteins, messenger RNA, microRNA, and bioactive lipids [12,16]. Key factors in the WJ-MSC secretome include VEGF, HGF, FGF-2, PDGF-AA, angiopoietin-1, EGF, TGF-β, GM-CSF, IL-10, and IGF-1. These factors contribute to angiogenesis, wound healing, immunomodulation, protection against apoptosis, and maintenance of cellular homeostasis [12,17,19]. The secretome’s capacity to modulate inflammation, stimulate cell proliferation, and promote tissue survival has encouraged its therapeutic use across multiple pathologies [12,17,19].

Cell-free therapy uses the secretome or its fractions instead of live cells. However, for cell-free therapy to be clinically viable, it requires a delivery system that ensures the stability and sustained bioavailability of bioactive factors at the site of action, preventing degradation and maintaining effective concentrations [17]. PLGA/NMP-based ISFI provides a robust technological platform by encapsulating and prolonging the release of WJ-MSC supernatant. Although PLGA/NMP-based ISFI systems offer technological advantages as carriers for WJ-MSC secretome, the use of PLGA/NMP as a parenteral excipient requires cytotoxicity evaluation, especially since multiple studies have mentioned cytotoxic effects when used in the construction of ISFI [10,20]. Therefore, the objective of this study was to evaluate the in vitro cytotoxicity, cell viability, and in vivo biocompatibility of PLGA/NMP-based ISFI loaded with WJ-MSC supernatant, and to determine whether incorporation of this cell-free bioactive fraction enhances the biological performance and local tolerability of the polymeric depot, thereby improving the safety-to-functionality balance of the sustained-release platform.

## 2. Materials and Methods

### 2.1. Materials

*N*-Methyl-2-pyrrolidone (NMP), a pharmaceutical-grade solvent (Pharmasolve^®^) was kindly donated by Ashland México (Ciudad de México, México). The PLGA grades used in this study were pharmaceutical-grade Viatel™ polymers kindly gifted by Ashland Mexico for research purposes. PLGA 50:50 was an acid-terminated polymer (lot 2471042), whereas PLGA 85:15 was also acid-terminated (lot 2485958). DMEM-F12, RPMI-1640, fetal bovine serum (FBS), and penicillin/streptomycin were purchased from Capricorn Scientific (Ebsdorfergrund, Germany). Calcein AM and Calcein Violet AM (Invitrogen™ C1430, Waltham, MA, USA), Ethidium Homodimer-1 (Invitrogen™ EthD-1, E1169, Waltham, MA, USA), PrestoBlue reagent (Invitrogen™ A13261, Waltham, MA, USA), Annexin V-Pacific Blue conjugate, and 7-AAD were obtained from Invitrogen™ (Waltham, MA, USA)/BioLegend (San Diego, CA, USA). The LegendPlex™ Human Growth Factor Panel was acquired from BioLegend (San Diego, CA, USA). All other reagents were analytical grade.

### 2.2. WJ-MSC Supernatant Preparation

Supernatant was obtained according to Jarquín-Yáñez K. et al., 2025 [19]. Briefly, WJ-MSCs were isolated from human umbilical cords via the explant method using DMEM-F12 supplemented with 10% FBS and 1% penicillin/streptomycin in a 37 °C incubator with 5% CO_2_. All procedures involving human tissue samples were approved by the Ethics Committee of the Faculty of Medicine, Universidad Nacional Autónoma de México (No. FM/DI/112/2022), approval date on 11 April 2023, with informed consent signed. Upon reaching 80% confluence, cells were preconditioned with 10 ng/mL EGF and 40 ng/mL FGF [19], washed, and cultured in serum-free medium to collect supernatant. The secretome was evaluated by flow cytometry (Attune NxT Invitrogen™, Waltham, MA, USA) using the LegendPlex growth factor panel (BioLegend, San Diego, CA, USA). Growth factors in WJ-MSC-conditioned medium were quantified using the LEGENDplex™ Human Growth Factor Panel (BioLegend, San Diego, CA, USA) according to the manufacturer’s instructions. Samples were incubated with capture beads and detection antibodies and analyzed by flow cytometry. Growth factor concentrations were calculated from the corresponding standard curves and expressed as pg/mL, ranging from 0 to ≥10,000 pg/mL, the upper limit of the assay range.

### 2.3. Preparation of PLGA/NMP ISFI Loaded with WJ-MSC Supernatant

ISFI formulations were prepared by dissolving 500 mg of PLGA (50:50 or 85:15) in 5 mL of NMP to achieve a polymer concentration of 100 mg/mL. The WJ-MSC secretome, obtained as a lyophilized powder, was weighed and incorporated directly as an anhydrous powder into the PLGA/NMP solution to a final concentration of 100 mg/mL (no aqueous phase was added). Table 1 describes the formulations evaluated.

Solutions were filtered through 0.22 µm Nylon membranes. All preparations were performed in a laminar-flow hood using sterile materials.

WJ-MSC was quantified as total protein by measuring absorbance at 280 nm [21] using a UV-Vis spectrophotometer (Shimadzu, model 1900, Kyoto, Japan); it was used as a bulk surrogate for overall payload release rather than for individual growth factors. Briefly, 500 µL of ISFI formulation was injected into 5 mL phosphate buffer (pH 6.8) at 37 °C to simulate physiological conditions. The assay was performed under static conditions, and all experiments were performed in triplicate. At each sampling time, the release medium was completely removed and replaced with fresh buffer. Released WJ-MSC-supernatant-derived material was estimated by absorbance at 280 nm using a calibration curve prepared with the same lyophilized WJ-MSC supernatant powder used in the formulations, within the range of 0.5–5.0 mg/mL (r^2^ = 0.9996). Cumulative release was calculated as $WJ-MSC\;\%=\left[\left(\sum C_{i}\;*\;V_{r}\right)/M_{0}\right]\;*\;100$, where $C_{i}$ is the concentration measured at each sampling interval after blank correction, $V_{r}$ is the replacement volume of release medium, and $M_{0}$ is the initial amount of lyophilized WJ-MSC supernatant powder loaded into the formulation. The graph was created using Origin 9.0.

### 2.4. Cell Viability and Cytotoxicity

All animal procedures were conducted in accordance with institutional guidelines and approved by the Institutional Animal Care and Use Committee (CICUAL, protocol no. 020-CIC-2025) of the Faculty of Medicine, Universidad Nacional Autónoma de México, approval date on 3 February 2026.. Two methodologies were employed with different cell strains. First, spleens from C57BL/6 mice (*n* = 3) from the Faculty of Medicine bioterium (Universidad Nacional Autónoma de México) were harvested in PBS, washed three times under sterile conditions, placed on a sterile mesh in a Petri dish with 3 mL RPMI, and mechanically triturated using a syringe plunger. The splenocyte suspension was collected in a 50 mL Falcon tube, centrifuged at 1800 rpm for 5 min, and seeded at 1 × 10^6^ cells/mL in 24-well plates pre-loaded with 100 µL PLGA (50:50) or PLGA (85:15), with or without 100 mg/mL WJ-MSC supernatant and untreated cells (without PLGA or PLGA with WJ-MSC supernatant). Cultures were maintained for 24 h in RPMI-1640 supplemented with 10% FBS and 1% penicillin/streptomycin at 37 °C with 5% CO_2_. Cells were then harvested, washed, resuspended in 100 µL of binding buffer in Eppendorf tubes, stained with 5 µL Annexin V-Pacific Blue and 5 µL 7-AAD, and incubated for 15 min at room temperature in the dark. After washing, all experiments were incubated in 100 µLwith 1 µL of calcein Violet AM for 15 min at room temperature in the dark. Subsequently, 400 µL of staining buffer was added, and samples were analyzed on an Attune NxT flow cytometer (National Flow Cytometry Laboratory, Institute of Biomedical Sciences, UNAM). Pacific Blue (405 nm excitation, ~450 nm emission) and 7-AAD (488 nm excitation, ~647 nm emission), and Calcein Violet AM (405 nm excitation, ~460 nm emission) were detected, with ≥10,000 events acquired per sample. Cell populations were classified as Viable cells: (Annexin V^−^/7-AAD^−^), Early apoptosis (Annexin V^+^/7-AAD^−^), Late apoptosis/necrosis (Annexin V^+^/7-AAD^+^), and necrosis (Annexin V^−^/7-AAD^+^). The viable cell population, Calceín Violet AM positivity, was assessed as an additional indicator of cellular viability; flow cytometry data were analyzed using FlowJo (version 10.6). The gating strategy was applied sequentially to exclude debris and non-cellular events. Followed by selection of the lymphocyte population based on forward and side scatter area (FSC-A and SSC-A) parameters. Doublets were excluded by sequential gating based on FSC-A versus FSC-H (height). The population of interest was subsequently identified based on the expression of markers.

For the second methodology, human gingival fibroblasts (HGF-1; ATCC^®^ CRL-2014™, Manassas, VA, USA) were obtained from the American Type Culture Collection (ATCC) and maintained under standard culture conditions. Cells were used between passages 5; they were seeded at 1 × 10^4^ cells/mL in 24-well plates pre-loaded with 100 µL PLGA (50:50) or PLGA (85:15), with or without 100 mg/mL WJ-MSC supernatant, untreated cells (without PLGA or PLGA with WJ-MSC supernatant), and a positive control of cell death induced by H_2_O_2_ and PLGA (50:50) or PLGA (85:15) without cells. Cultures were maintained for 24 h and 4 days in DMEM supplemented with 10% FBS and 1% penicillin/streptomycin at 37 °C with 5% CO_2_. Cells were washed twice with Hank’s Balanced Salt Solution (HBSS) and stained with 0.1 µL Calcein AM (Invitrogen™ C1430, Waltham, MA, USA) and 1 µL Ethidium Homodimer-1 (Invitrogen™ E1169, Waltham, MA, USA) in 500 µL HBSS for 30 min at 37 °C. Samples were imaged using a Nikon Eclipse Ti2 microscope (4×, 10×, 20× objectives) with NIS-Elements software, AR, version 7.00.00 (Nikon, Tokio, Japan).

### 2.5. Cell Proliferation

Human fibroblasts (HGF-1; ATCC^®^ CRL-2014™, Manassas, VA, USA) were seeded at 1 × 10^4^ cells/well in 96-well plates pre-loaded with 10 µL PLGA (50:50) or PLGA (85:15), with or without 100 mg/mL WJ-MSC supernatant and untreated cells (without PLGA or PLGA with WJ-MSC supernatant). Cultures were maintained for 24 h and 4 days in DMEM supplemented with 10% FBS and 1% penicillin/streptomycin at 37 °C with 5% CO_2_. Cells were washed with HBSS, and 10 µL PrestoBlue reagent (Invitrogen™ A13261, Waltham, MA, USA) plus 90 µL HBSS was added, incubated for 1 h at 37 °C with 5% CO_2_, and absorbance was read at 570 nm.

### 2.6. In Vivo Biocompatibility

Eight-week-old C57BL/6 mice (*n* = 3) from the Faculty of Medicine bioterium (UNAM) received subcutaneous injections (ventral left caudal flank) of 100 µL PLGA (50:50) or PLGA (85:15), with or without 100 mg/mL WJ-MSC supernatant. At 14 days post-injection, injection sites were evaluated via conventional histological processing and H&E staining.

### 2.7. Statistical Analysis

The significance level was set at *p* < 0.05. Differences among groups were analyzed using one-way ANOVA, followed by subsequent analysis of the means using Dunnett’s post hoc test for multiple comparisons with the control group as the reference. All data were analyzed statistically using Minitab 19 software.

## 3. Results and Discussion

### 3.1. WJ-MSC Supernatant Characterization

Analysis of the WJ-MSC supernatant using the LegendPlex™ panel quantified 13 growth factors in WJ-MSC samples derived from three independent umbilical cord donors (*n* = 3), with concentrations ranging from 0 to ≥10,000 pg/mL. Data were presented as mean ± standard deviation and coefficient of variation (Table 2). The highest concentrations of growth factors were observed in HGF (≥10,000 pg/mL) corresponding to the upper limit of the assay range and Ang-1 (9366 pg/mL), while the lowest concentrations were in TGF-α (1240.5 pg/mL), GM-CSF (1554 pg/mL) and G-CSF (1655.82 pg/mL) (Figure 1). This bioactive factor profile aligns with the well- established paracrine activity of WJ-MSCs, encompassing potent angiogenic (VEGF, angiopoietin-1), mitogenic (EGF, PDGF-AA), and immunomodulatory (TGF-α, M-CSF) stimuli involved in tissue repair, cell survival, and immune response modulation [12,17]. The biological variability of growth factors was assessed using the coefficient of variation (CV%). CV ranged from 0% to 68.6%, according to the interpretation guidelines, HGF and EGF showed high homogeneity between samples (CV 0–0.4%), Ang-1, PDGF-AA and VEGF (CV < 15%) had low variability, for TGF-α, M-CSF, PDGF-BB, FGF and EPO the variability was moderate (CV 15–30%), while GM-CSF showed high variability (CV 30–50%), finally the factors G-CSF and SCF showed marked heterogeneity between samples (CV > 50%) (Table 2).

In general, 10 of the 13 growth factors, representing 76.9%, showed a coefficient of variation (CV) < 30%, indicating low/moderate variability and suggesting that most growth factors were relatively homogeneously distributed among the samples, although they retained a certain degree of biological variability, as expected in human samples. On the other hand, only 3 growth factors showed significant dispersion (CV > 50%), reflecting biological heterogeneity among individuals and highlighting the existence of sensitive interindividual biomarkers, a phenomenon previously described by Mithal, L.B. et al., 2025 [22] in studies of umbilical cord blood and other perinatal tissues, in which the blood group plays an important role in factor expression, and Romanov, Y.A. et al., 2019 [23] reported that the physiological state of newborns throughout gestational age was crucial for proteome variability. Also, angiopoietin-1 and VEGF promote angiogenesis/vascular stabilization, which is essential for implant-tissue integration [12]. EGF and PDGF-AA stimulate fibroblast proliferation/migration, favoring organized capsule formation over excessive fibrosis [24]. The immunomodulatory growth factors (TGF-β, M-CSF, inferred IL-10) shift macrophages from pro-inflammatory M1 to reparative M2 phenotypes [17,25]. In the present study, M-CSF was specifically quantified in the WJ-MSC supernatant, supporting its potential involvement in the immunomodulatory properties of the formulation. However, TGF-β and IL-10 were not directly evaluated in this study, and their contribution to the observed effects cannot be established. Further studies assessing these and other immunomodulatory factors will be necessary to elucidate the mechanisms underlying the biological effects of the WJ-MSC supernatant. Therefore, the growth factors commonly found in MSC supernatant are involved in inducing healing of lesions of diverse origin.

### 3.2. WJ-MSC Supernatant and Release Evaluation

The PLGA concentration of 100 mg/mL in NMP was selected based on previous ex-perience from our group on PLGA/NMP in situ-forming systems. Under the present ex-perimental conditions, this concentration allowed complete polymer dissolution, ade-quate injectability, suitable apparent viscosity, and visible in situ depot formation after contact with aqueous medium. Since this is a first proof-of-concept study combining PLGA/NMP ISFI with WJ-MSC supernatant, this concentration was selected as a prelimi-nary formulation platform to evaluate release behavior, cytocompatibility, local tissue response, confirming clinical applicability and providing data to refine formulations.

PLGA 50:50 and PLGA 85:15 were selected to compare polymeric matrices with contrasting expected degradation and release behaviors: PLGA 50:50, with higher glycolide content and faster hydration/degradation, and PLGA 85:15, with higher lactide content and slower water uptake and release. Figure 2 depicts the cumulative release profile of WJ-MSC supernatant from ISFI formulated with PLGA 50:50 and PLGA 85:15. Both systems exhibited an initial burst phase during the first hours, characteristic of phase-inversion systems where solvent-water exchange precipitates the polymer and concomitantly releases soluble WJ-MSC supernatant [5,26]. Significant differences emerged in burst magnitude and subsequent kinetics between formulations. PLGA 50:50 showed higher cumulative release throughout the study, reaching 29.80 ± 2.57% at 4 h, 45.56 ± 1.78% at 25 h, and 55.26 ± 2.10% at 54 h. In contrast, PLGA 85:15 displayed a slower release profile, reaching 26.24 ± 2.77% at 4 h, 36.00 ± 1.74% at 25 h, and 40.59 ± 2.05% at 54 h. This differential behavior stems from hydrophobicity differences; the higher lactic acid content in PLGA 85:15 imparts greater hydrophobicity, retarding water ingress and supernatant diffusion [9,27]. These findings corroborate prior studies demonstrating that the lactide:glycolide ratio is a critical determinant of PLGA-based release kinetics, with increased lactic acid proportion enhancing matrix stability and attenuating initial release [27,28]. When creating an in situ-forming implant, it is crucial to evaluate how additives in the final formulation can influence the secretome release profile. Understanding the role of the polymer that governs the release mechanism is essential before delving into the effects of these additives. This knowledge enables more predictable and effective implant design, ensuring the intended therapeutic effects are achieved in a controlled manner. These findings suggest that PLGA 50:50 formulation with the WJ-MSC supernatant may mitigate early cytotoxicity associated with NMP exposure.

### 3.3. Cell Viability and Cytotoxicity

The effect of PLGA/NMP on apoptosis was assessed by flow cytometry using dual Annexin V-Pacific Blue/7-AAD staining to evaluate viability (Annexin V^−^/7-AAD^−^), early apoptosis (Annexin V^+^/7-AAD^−^), late apoptosis (Annexin V^+^/7-AAD^+^), and necrosis (Annexin V^−^/7-AAD^+^) in murine splenocytes exposed to ISFI formulations for 24 h. Analysis of the cell population revealed primarily viable cells and cells in early apoptosis, with no population corresponding to late apoptosis or necrosis observed. Subsequently, the cells classified as live were analyzed using calcein staining to assess their viability and confirm their metabolically active state. Figure 3 shows representative dot plots (A) Gating of the population of interest; (B) Singlets; (C) Unstained cells (s/t); (D) untreated cells; (E) PLGA 50:50/NMP; (F)PLGA 50:50/NMP + WJ-MSC supernatant; (G) PLGA 85:15/NMP; (H) PLGA 85:15/NMP + WJ-MSC; Calcein fluorescence histograms (I) untreated cells; (J) PLGA 50:50/NMP; (K) PLGA 50:50/NMP + WJ-MSC supernatant; (L) PLGA 85:15/NMP; (M) PLGA 85:15/NMP + WJ-MSC supernatant from three independent experiments, and (N) viable cells from the treatments (Data presented as mean ± standard deviation). It revealed that PLGA 50:50 + WJ-MSC supernatant group reached a cell viability of 81.2% (Figure 3F,N), which was statistically significant (*p* < 0.05) compared with the untreated control (77.2%, Figure 3D) and PLGA 50:50 (76.6%, Figure 3E). Conversely, PLGA 85:15 (73.8%) and PLGA 85:15 + WJ-MSC supernatant (72.4%) groups showed significantly reduced viability (Figure 3F–H), suggesting an early cytotoxic effect associated with PLGA/NMP exposure during implant formation [9,10]. This suggests slower supernatant release from PLGA 85:15 fails to deliver sufficient immediate cytoprotection against NMP toxicity, rendering splenocytes more apoptosis-prone in the first 24 h. This finding matters because it drives the release profile needed for the secretome to promote favorable tissue regeneration. Consistent with our findings, 81.2% splenocyte viability for PLGA 50:50 + supernatant, a study reported that intravenous human MSCs in a cecal ligation/puncture (CLP) sepsis mouse model markedly reduced pro-inflammatory cytokines (TNF-α, IL-6, IL-1β), elevated IL-10, preserved spleen morphology, and decreased splenic lymphocyte apoptosis [29].

The cytoprotective effect observed with PLGA 50:50 + supernatant is consistent with the previously described ability of the MSC secretome to modulate cell survival via soluble mediators such as IL-10, HGF, and TGF-β [17,25]. Previous studies have also linked the MSC secretome to reduced apoptotic responses and the modulation of intracellular survival pathways, including the PI3K/AKT pathway [17,30]. However, these mechanisms were not directly investigated in the present study; consequently, it is not possible to determine the contribution of specific growth factors or intracellular signaling pathways to the observed cytoprotective effect. The rapid availability of soluble factors from the PLGA 50:50 could be relevant for splenocytes, given their short half-life [24]. Furthermore, it is worth noting that the NMP dose did not appear to be the cause of the induction of the various types of cell death, as the control group exhibited a higher percentage of non-viable cells than the other experimental groups.

Studies suggest that the therapeutic effect of MSCs depends not only on the induction of survival but also on mechanisms associated with apoptosis and the release of bioactive paracrine factors. In this regard, a recent investigation describes how apoptotic MSCs release extracellular vesicles and mediators that modulate inflammation, reduce apoptosis, and promote cell survival through associated IL-10, TGF-β, and PI3K/AKT pathways [31]. In addition, it has been reported that controlled-release biomaterial systems can improve the stability and bioavailability of the MSC-derived secretome, thereby promoting survival of up to 71.7% and modulating the local tissue response [32]. Consistent with this, our results show that these biomaterials do not function merely as passive vehicles but rather generate a microenvironment that provides prolonged, sustained protection of bioactive factors for up to 1 month.

Cell viability was assessed via Calcein AM (live cells, green fluorescence) and Ethidium Homodimer-1 (dead cells, red fluorescence) staining at 24 h and 4 days. Note that the polymer turns orange. At 24 h (Figure 4, upper panel), PLGA 50:50 + WJ-MSC supernatant displayed the highest number of viable cells, compared to cells treated with PLGA 50:50 and PLGA 85:15 + supernatant. This trend persisted for 4 days (lower panel), corroborating superior viability with PLGA 50:50 + supernatant and suggesting bioactive factor release.

Conversely, groups without supernatant or containing PLGA 85:15 + supernatant showed a predominance of EthD-1–positive cells and a lower proportion of calcein-AM–positive cells, indicating loss of membrane integrity and reduced cell viability. These results reinforce the notion that early release of trophic factors from PLGA 50:50 is essential for fibroblast survival during critical implant formation.

Additionally, PLGA 50:50 and 85:15 without cells, as other controls subjected to the same calcein staining protocol, were analyzed. Staining associated with the polymers was observed even in the absence of cells, indicating that the material exhibited a fluorescent signal under the conditions evaluated; however, this signal is not confused with the cells. (Figure 4B).

It is important to emphasize the significance of fibroblast cytotoxicity assays. Fibroblasts play a pivotal role in connective tissue response to implants, mediating collagen synthesis, extracellular matrix remodeling, and fibrotic capsule formation [24]. Supernatant-mediated preservation of fibroblast viability suggests that bioactive factors promote controlled, biocompatible encapsulation.

In this study, the 50:50 PLGA formulation with WJ-MSC supernatant was associated with reduced cytotoxicity under NMP conditions. Previous research has reported that MSC-derived extracellular vesicles can inhibit macrophage activation through suppression of NF-κB/MAPK signaling, thereby reducing the release of inflammatory mediators such as IL-6 and IL-1β [33]. Although these mechanisms were not directly evaluated in the present study, the observed reduction in cytotoxicity suggests that WJ-MSC supernatant may contribute to a more favorable cellular microenvironment.

### 3.4. Metabolic Activity

Figure 5 illustrates fibroblast metabolic activity via PrestoBlue assay (resazurin absorbance at 570 nm) at 1 and 4 days. PLGA 50:50 + supernatant matched the untreated control in viability (no significant difference), whereas the other groups showed a significant population decline compared with both the untreated control and PLGA 50:50 + supernatant. At 24 h, PLGA 50:50 + supernatant absorbance matched the control (*p* > 0.05), indicating effective mitigation of initial PLGA/NMP cytotoxicity. At 4 days, it exceeded the control (*p* < 0.05), suggesting sustained proliferative stimulation via secretome mitogens (EGF, PDGF-AA, VEGF) that activate the PI3K/AKT and MAPK/ERK pathways [17,24]. Formulations without supernatant showed significantly lower absorbance at both time points (*p* < 0.01) compared with the control group, attributable to residual NMP toxicity. PLGA 85:15 + supernatant exhibited partial recovery but did not reach control levels due to slower payload release. PrestoBlue detects cell-mediated cytotoxicity in vitro by measuring resazurin reduction by mitochondrial enzymes (Figure 5). Changes in color, as well as shifts in fluorescence, can be quantified using either a fluorometric or a spectrophotometric approach [34]. Results confirm PLGA 50:50 + WJ-MSC supernatant as optimal for maintaining/stimulating fibroblast metabolism, a key biocompatibility metric.

### 3.5. In Vivo Biocompatibility

Histological evaluation of the application site 14 days after subcutaneous implantation revealed the body’s tissue response (Figure 6). Untreated controls showed normal architecture of the epidermis, dermis, and subcutaneous tissue; connective tissue and collagen fibers were organized with no signs of inflammation (Figure 6A,B). In contrast, in C57BL/6 mice injected with ISFI (Figure 6C–F), the material was identified as acellular eosinophilic material surrounded by acute and chronic inflammation. The inflammatory response was qualitatively assessed as mild, moderate, or severe: mild if there were scattered clusters of inflammatory cells without confluence; moderate when there were distinct, confluent clusters of inflammatory cells; and severe when the inflammatory infiltrate was pronounced and may alter the tissue architecture itself.

In assays with PLGA 50:50/NMP (without supernatant), the application of ISFI induced fibrosis and an inflammatory response consisting of macrophage infiltration, moderate lymphocytes, and few polymorphonuclear leukocytes. In Figure 6C, no multinucleated giant cells or adjacent necrosis were observed, which is consistent with the biocompatibility of PLGA/NMP [9].

In histological sections with PLGA 50:50 + WJ-MSC supernatant (Figure 6E), a minor fibroblastic reaction was identified; a reduced amount of ISFI was observed, accompanied by a mild mixed inflammatory response, made up of macrophages, lymphocytes, and a few polymorphonuclear cells, which suggests that secretome immunomodulators (IL-10, TGF-β, HGF) accelerate resolution/remodeling [17,25]. The PLGA 85:15 groups (Figure 6D,F) exhibited a fibroblastic reaction comparable to the PLGA 50:50 groups (Figure 6C,E); however, they showed moderate chronic inflammation, and the acute inflammatory component was slightly more pronounced.

The PLGA 85:15 + WJ-MSC supernatant (Figure 6F) groups showed a very similar inflammatory response to the group without supernatant (Figure 6D), with a greater polymorphonuclear component; however, in the image (Figure 6E), we can observe that the level of application is deeper, finding skeletal muscle fibers immersed, which leads to a more pronounced inflammatory response by the surrounding tissues with respect to the application site.

In none of the groups compared were necrosis, abscesses, or severe inflammation found, thus meeting the criteria for local effects in ISO 10993-6:2016 standard [35].

Through histopathological analysis of the different groups, the PLGA 50:50 groups (±supernatant) appeared to exhibit a more favorable tissue response and faster remodeling. An inflammatory response was observed in all groups, though it was less pronounced in the PLGA 50:50 formulations. However, this finding should be interpreted with caution, as local inflammatory changes may be influenced by the implantation site rather than the ISFI itself. Furthermore, no signs of persistent or severe chronic inflammation, the appearance of foreign body giant cell formation, necrosis, or other signs of severe tissue reaction was observed in any group.

A recent study reported that mesenchymal stem cells can inhibit macrophage activation by suppressing the NF-κB and MAP kinase pathways, thereby reducing the release of inflammatory mediators such as IL-6 and IL-1β [33]. While these mechanisms were not directly evaluated in the present study, our findings suggest that WJ-MSC supernatant may contribute to mitigating NMP-associated cytotoxicity and may influence the cellular microenvironment, potentially supporting a more favorable cellular response.

A wide variety of bioimplants composed of both natural and synthetic polymers are available on the market for dermal implantation. These bioimplants elicit a cellular immune response, including macrophage activation, which is expected and well-documented in this type of implant [36]. Currently, other types of bioimplants exist, such as ISFI, which are used as vehicles for the controlled and sustained release of drugs, including chemotherapeutic agents and antibiotics. Examples include Lupron Depot^®^, Eligard^®^, and others created from PLGA and other copolymers for osteosynthesis. These bioimplants generally exhibit histological reactions consisting of macrophage activation, cellular debris, endothelial activation, fibrosis, infiltration by plasma cells, lymphocytes, and polymorphonuclear leukocytes, with evidence of old hemorrhage and, in other cases, multinucleated giant cells with a foreign body reaction. They can present adverse reactions with visible clinical repercussions such as swelling, redness, and pain, which were controlled with analgesics, steroids, and heat therapy, with no serious complications reported. In general, tissue degradation and biocompatibility will depend on the different implanted materials, their molecular structure, volume, application site, and design quality, as well as the various comorbidities present in patients [37,38].

## 4. Limitations, Challenges, and Future Perspectives

Although this study established a standardized protocol for obtaining and processing WJ-MSC supernatant and evaluated the levels of specific bioactive molecules from each umbilical cord, a certain degree of biological variability among donors, inherent to cell-derived secretome products, may persist. Factors related to the tissue donor’s biological characteristics and the intrinsic properties of the WJ-MSCs can influence the composition and relative abundance of the secreted bioactive factors. Consequently, further studies involving a larger number of donors and comprehensive secretome characterization would be valuable to define this variability and identify critical quality attributes associated with biological potency. Nevertheless, the standardized procedures and the characterization of specific molecules from each cord implemented in this study provide an important foundation for enhancing the reproducibility and consistency of the WJ-MSC supernatant used in the PLGA/NMP formulation [17,39].

On the other hand, the 14-day in vivo evaluation represents a limitation of the present study; this experimental period was deliberately chosen as a preliminary assessment to evaluate the early biological response and cytotoxicity of the various formulations. This approach allowed for the comparison of their initial in vivo behavior and the establishment of a rational basis for selecting the formulation with the most favorable profile for future research. Thus, this study constitutes an initial step toward the development of a PLGA/NMP implant loaded with WJ-MSC supernatant [10,36], although longer-term studies will be required to determine the durability of the response, implant degradation, the release kinetics of bioactive factors, and delayed local and systemic effects. Furthermore, disease-specific animal models should be used to determine the therapeutic relevance of this delivery approach. A potential limitation of subcutaneous administration is the slight variability in injection depth resulting from anatomical differences in the dorsal subcutaneous tissue of the mice; however, the consistency of the immunological response observed across the animals suggests that this variability did not substantially influence the overall findings. Future studies should further standardize the administration procedure by implementing more precise control over injection depth and confirming implant localization, in order to minimize procedural variability.

## 5. Conclusions

This study demonstrates that the PLGA/NMP ISFI platform can provide controlled release of WJ-MSC supernatant, provided that polymer composition and release kinetics are appropriately optimized. Notably, PLGA 50:50 + supernatant excelled, showing superior splenocyte viability/low apoptosis (Annexin V/7-AAD), and fibroblast preservation/proliferation stimulation (Calcein AM/EthD-1, PrestoBlue). These consistent results indicate that early, sufficient release of trophic/immunomodulatory factors is critical for counteracting NMP-induced cytotoxicity in sensitive cells.

In vitro release data confirm faster early release for PLGA 50:50 than for 85:15, translating into greater early secretome availability, whereas the more hydrophobic 85:15 released more slowly. Therefore, the lactide:glycolide ratio governs the balance between availability and duration and should be matched to the target pathophysiology and predominant cell type.

In vivo histology complements these, confirming PLGA subcutaneous biocompatibility) and supernatant-associated thinner capsules/moderate inflammation, enabling adequate tissue integration. Correlative evidence suggests WJ-MSC supernatant harbors sufficiently active factors to elicit immunomodulatory/antiapoptotic effects sans live cells.

Collectively, the findings endorse cell-free therapy via PLGA 50:50/NMP ISFI as a potential strategy for site-specific modulation of viability and immunocompatibility without live-cell transplantation. Long-term studies and disease-specific models are warranted to validate sustained effects and advance clinical translation for prolonged trophic factor delivery in systemic inflammation/immunodamage pathologies. The present study demonstrates that the PLGA 50:50 formulation loaded with WJ-MSC supernatant represents the most suitable option for developing a biocompatible controlled-release system.

## Figures and Tables

**Figure 1:**
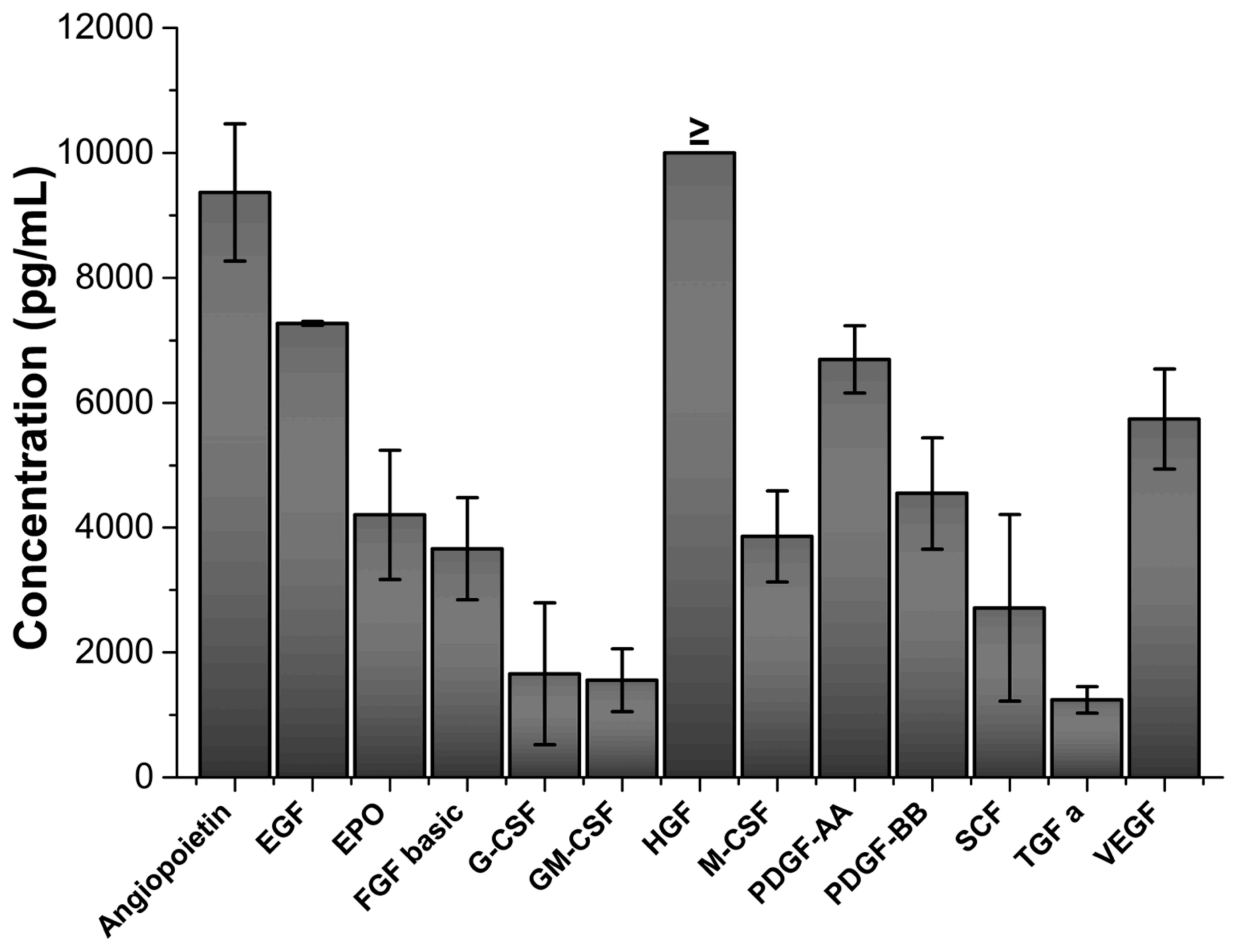
Graph of the quantification of growth factors found in the mesenchymal cell supernatant of Wharton’s jelly using the LegendPlex growth factor panel (BioLegend). Data are presented as mean ± SD (*n* = 3). HGF concentrations reaching the upper limit of the standard curve are indicated as ≥10,000 pg/mL. All other growth factor concentrations were within the quantifiable range of the assay.

**Figure 2:**
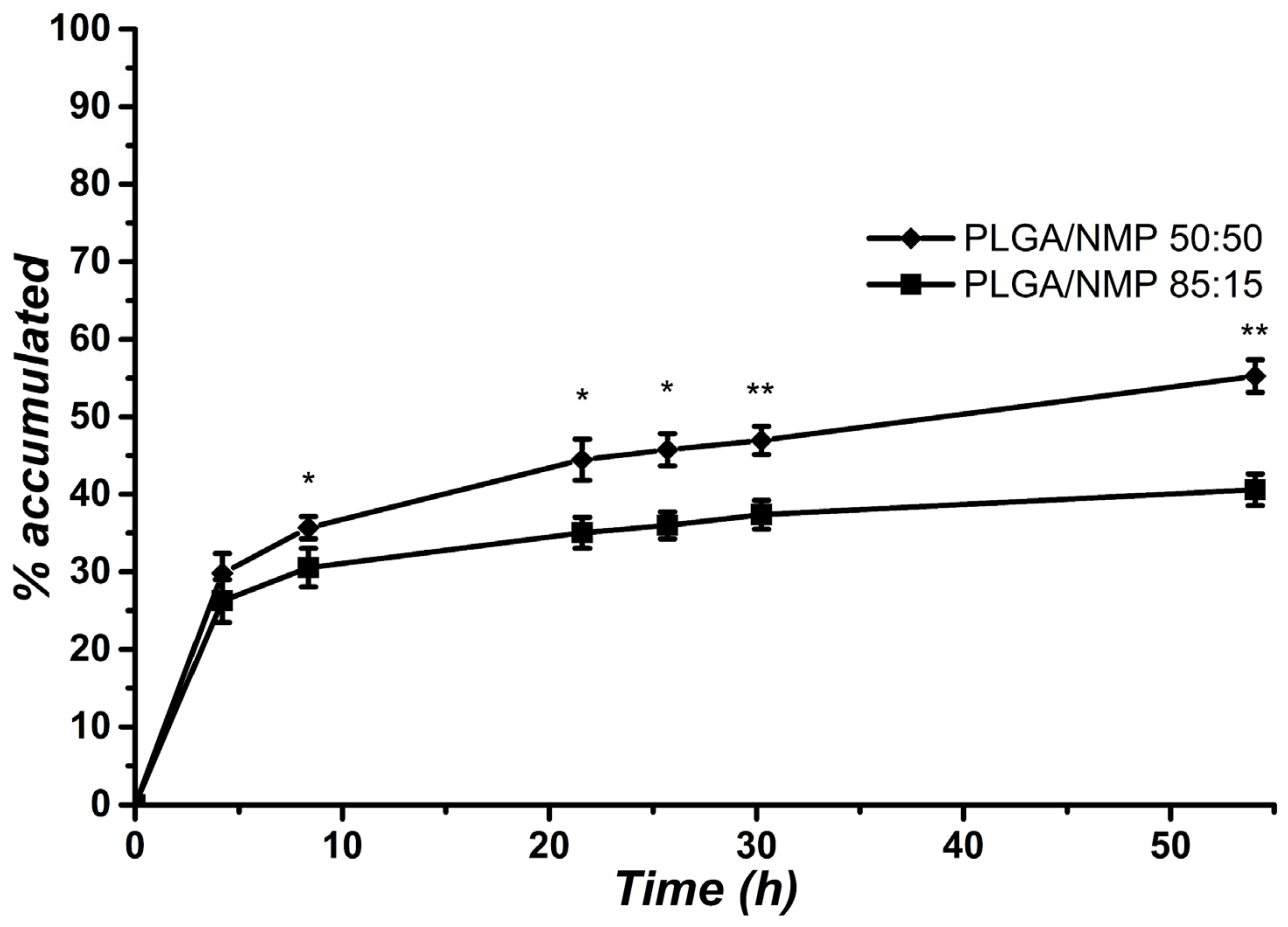
Cumulative release of WJ-MSC supernatant from PLGA/NMP in situ-forming implants with PLGA 50:50 (♦) and PLGA 85:15 (■). Data are presented as mean ± SD (*n* = 3). Statistical differences between formulations at each time point were analyzed by two-way ANOVA * *p* < 0.05; ** *p* < 0.01. Symbols in the legend correspond to the plotted curves.

**Figure 3:**
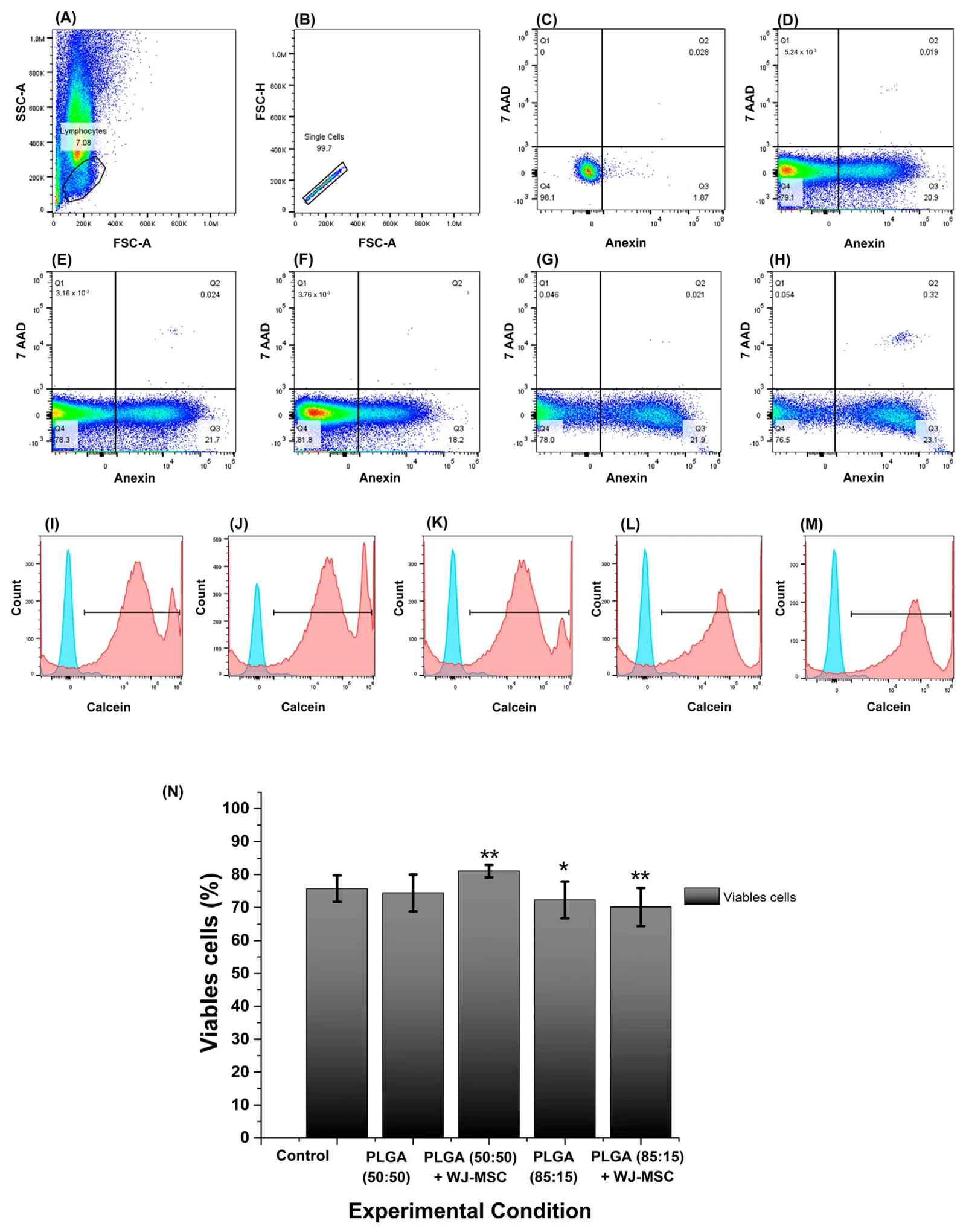
Dot plots: (**A**) Gating of the population of interest; (**B**) Singlets; (**C**) Unstained cells (s/t); (**D**) untreated cells; (**E**) PLGA 50:50/NMP; (**F**) PLGA 50:50/NMP + WJ-MSC supernatant; (**G**) PLGA 85:15/NMP; (**H**) PLGA 85:15/NMP + WJ-MSC supernatant; Calcein fluorescence histograms: (**I**) untreated cells; (**J**) PLGA 50:50/NMP; (**K**) PLGA 50:50/NMP + WJ-MSC supernatant; (**L**) PLGA 85:15/NMP; (**M**) PLGA 85:15/NMP + WJ-MSC supernatant from three independent experiments, and (**N**) Graphic of Annexin V/7-AAD. Data are presented as average ± SD (*n* = 3), * *p* < 0.05, ** *p* < 0.01.

**Figure 4:**
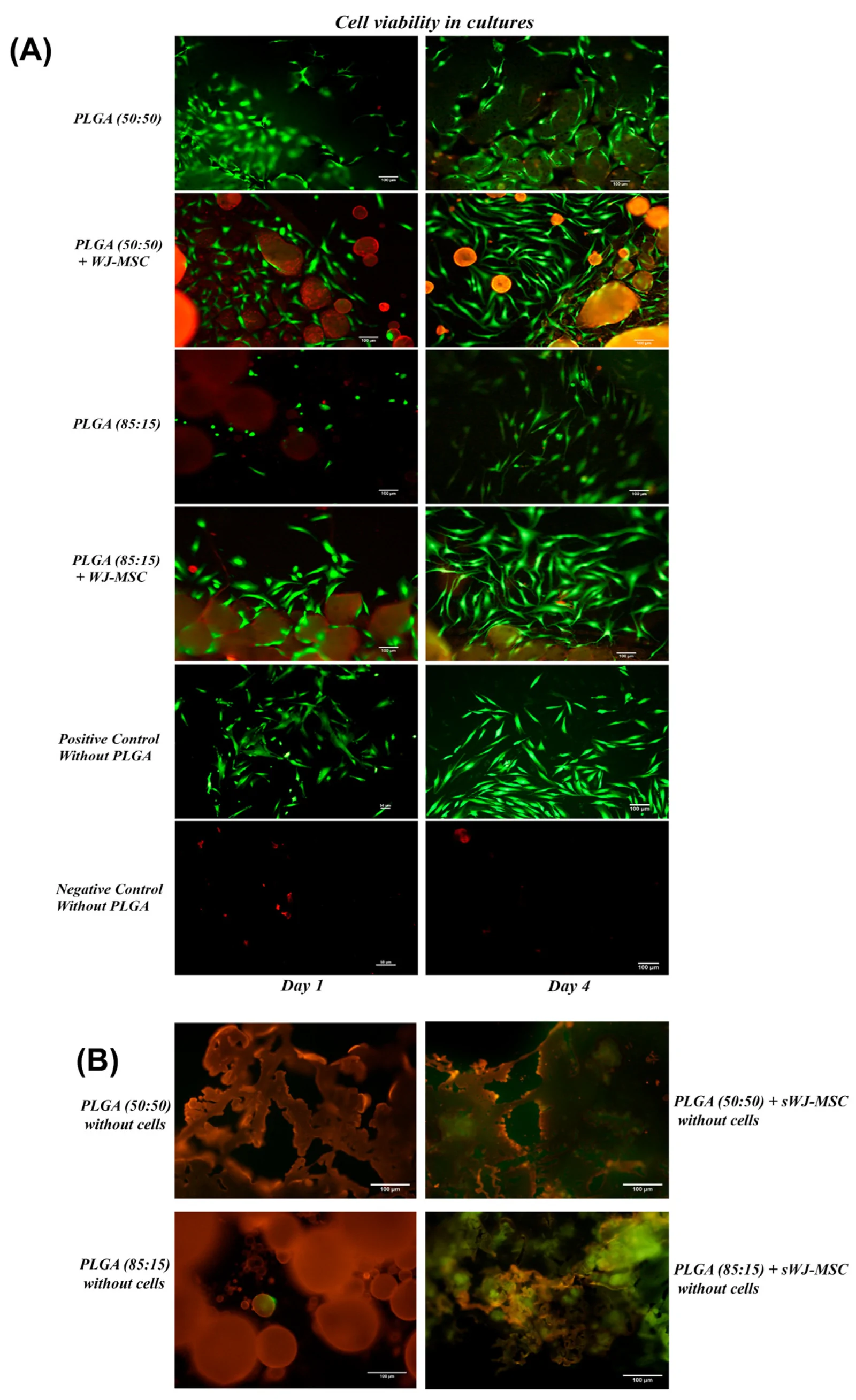
(**A**) Fluorescence photomicrographs of human fibroblasts stained with Calcein AM (green, live) and Ethidium Homodimer-1 (red, dead) after 24 h (upper) and 4 days (lower) exposure to PLGA/NMP ISFI. (**B**) Fluorescence photomicrographs of PLGA/NMP ISFI without cells. Scale bar: 100 µm.

**Figure 5:**
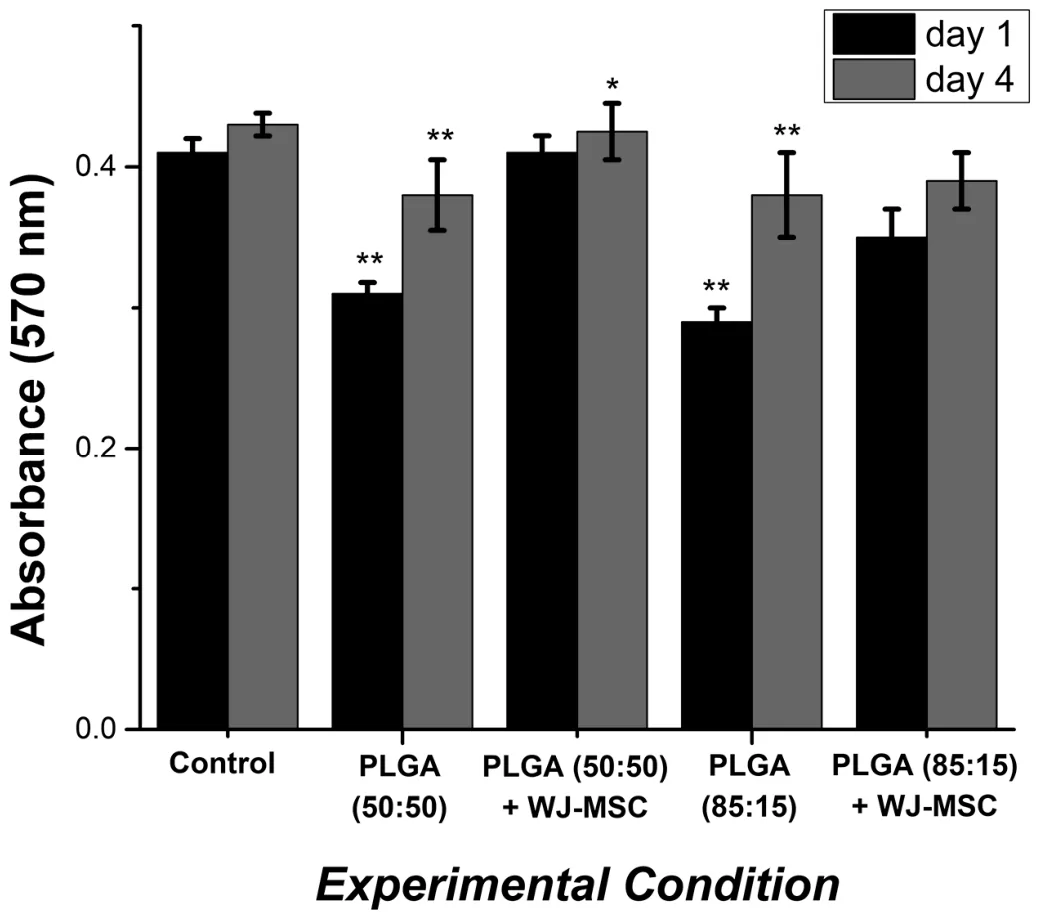
Metabolic activity of human fibroblasts via PrestoBlue after 1- and 4-day exposure to PLGA/NMP ISFI. Values: mean ± SD (*n* = 6). * *p* < 0.05, ** *p* < 0.01 vs. control (one-way ANOVA + Tukey’s test).

**Figure 6:**
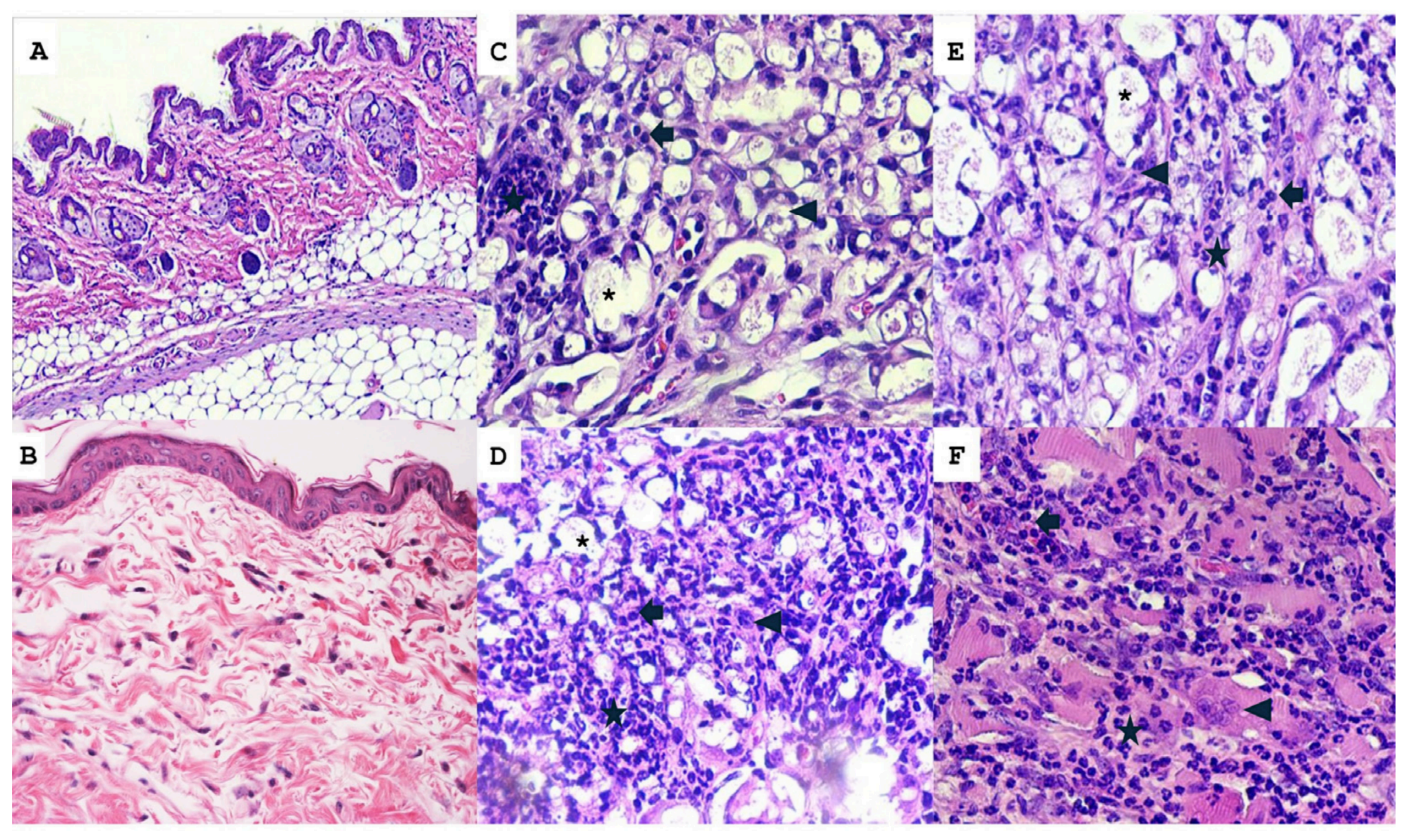
Representative H&E-stained micrographs of subcutaneous tissue 14 days after PLGA/NMP ISFI implantation. (**A**) The panoramic view shows a full-thickness section of rodent skin with preserved normal architecture, allowing for the visualization of the different layers and cutaneous adnexa; the epidermis exhibits keratinization, the dermal collagen fibers appear organized and homogeneous, the adnexa show a regular distribution, and the subcutaneous tissue appears organized (10×) and (**B**) a full-thickness section of rodent skin (40×). (**C**) PLGA 50:50/NMP (without supernatant). The ISFI remnants are visualized as acellular eosinophilic material surrounded by inflammatory infiltrates composed of lymphocytes, macrophages, and some polymorphonuclear leukocytes (40×). (**D**) PLGA 85:15 (without supernatant): The acute component, composed of neutrophil infiltrate, was greater than in the 50:50 groups with or without supernatant (40×). (**E**) PLGA 50:50 + WJ-MSC supernatant: a less pronounced fibroblastic reaction with a less pronounced inflammatory process was identified compared to the other groups (40×). (**F**) PLGA 85:15+ WJ-MSC supernatant: The inflammatory component was very similar to (**D**); however, striated skeletal muscle fibers were identified alternating with the inflammatory infiltrate (**F**), reflecting a variation in the anatomical depth of injection (40×). Markers: ISFI (asterisk), macrophages (arrowhead), lymphocytes (arrow), polymorphonuclear leukocytes (star).

**Table 1:** Formulations evaluated.

Formulation	PLGA Grade	PLGA Concentration	Solvent	WJ-MSC Secretome	Quantity/mL
PLGA 50:50/NMP	PLGA 50:50, acid-terminated	100 mg/mL	NMP	—	—
PLGA 50:50/NMP + WJ-MSC	PLGA 50:50, acid-terminated	100 mg/mL	NMP	Lyophilized WJ-MSC supernatant powder	100 mg/mL
PLGA 85:15/NMP	PLGA 85:15, acid-terminated	100 mg/mL	NMP	—	—
PLGA 85:15/NMP + WJ-MSC	PLGA 85:15, acid-terminated	100 mg/mL	NMP	Lyophilized WJ-MSC supernatant powder	100 mg/mL

**Table 2:** Growth factors quantified via LegendPlex™ panel in WJ-MSC supernatant.

Growth Factors	Concentration (pg/mL)	Standard Deviation (SD)	Coefficient of Variation (CV%)
Angiopoietin	9366	1097.9	11.7
EGF	7271	30.7	0.4
EPO	4204	1037.7	24.6
FGF basic	3659	817.4	22.3
G-CSF	1655.8	1135.9	68.6
GM-CSF	1554	502.5	32.3
HGF	≥10,000	0	0
M-CSF	3856.9	729.5	18.9
PDGF-AA	6695.9	538.7	8
PDGF-BB	4547	896.1	19.7
SCF	2713	1493.7	55
TGF-α	1240.5	212.1	17.1
VEGF	5743	799.8	13.9

## Data Availability

The original contributions presented in the study are included in the article, further inquiries can be directed to the corresponding authors.

## Abbreviations

The following abbreviations are used in this manuscript:

FSC-A	Forward Scatter Area
FSC-H	Forward Scatter Height
ISFI	In situ-forming implants
PLGA	Poly-(lactic-co-glycolic acid)
NMP	*N*-Methyl-2-pyrrolidone
MSCs	Mesenchymal stem cells
SSC-A	Side Scatter Area
SSC-H	Side Scatter Height
WJ-MSCs	Wharton’s jelly of the umbilical cord
